# A survey on the attitudes of Chinese medical students towards current pathology education

**DOI:** 10.1186/s12909-020-02167-5

**Published:** 2020-08-08

**Authors:** Chun Xu, Yiping Li, Pingsheng Chen, Min Pan, Xiaodong Bu

**Affiliations:** Department of Pathology and Pathophysiology, School of Medicine, Southeast University, Nanjing, 210009 China

**Keywords:** Pathology education, Traditional and online courses, Professional choice, Survey, Chinese medical students

## Abstract

**Background:**

Pathology education provides information on pathology and guides students to become pathologists. Recently, the Ministry of Education of the People’s Republic of China required the establishment of the system of ‘High-quality Online and Offline Courses’, which indicates that online courses will play an important role in higher education. Furthermore, the number of pathologists currently cannot satisfy clinical needs. To solve this health issue and implement the policy from the Ministry of Education, it is necessary to improve the current state of pathology education. First, we need to know students’ opinions of the current courses and their professional choices.

**Methods:**

Online questionnaires covering the quality of traditional courses, attitudes towards online courses, and suggestions for optimizing courses were designed and applied. Whether students want to become pathologists and the underlying reasons for this interest are also included in this survey. Participants are medical students from certain colleges in Nanjing. The collected data were assessed by statistical analyses, and *p*-values less than 0.05 were considered significant.

**Results:**

Of the 342 valid responses, 60.94% of undergraduate students showed their interest in pathology courses, and among them, 48.72% expressed that they may become pathologists. However, the corresponding percentage is only 29.59% in the group without interest. To optimize curricula, the top two suggestions are introducing more clinical cases (undergraduate students, 64.45%; graduate students, 79.09%) and making the classes lively and interesting (undergraduate students, 59.77%; graduate students, 62.79%). Approximately 80.00% of students consider online courses to be good supplementary materials to traditional courses, and approximately half prefer an online-offline mixed learning model. Salary, interest, and employment status are the main factors influencing students’ professional choices.

**Conclusions:**

Students are generally satisfied with traditional pathology courses, and online courses are good supplementary materials in their opinions. It has been suggested that clinical cases be introduced in classes. It is more likely that students who have an interest in pathology will become pathologists. The data from this survey also show that the main causes of the shortage of pathologists are a lack of engaging work and an unsatisfactory salary.

## Background

To improve teaching quality, promote a revolution in classroom teaching, and explore new forms of intelligent education, the Ministry of Education of the People’s Republic of China has started a curriculum reform called ‘High-quality Online and Offline Courses’. [1] This policy creates both new opportunities and new challenges for higher education. Optimizing traditional courses, establishing online courses, and rationally integrating the two are important tasks of modern university teachers.

Pathology is a discipline bridging basic medicine and clinical medicine. Therefore, it is a compulsory course for medical students. Although online pathology courses are available on some platforms of network education, they mainly serve as materials for medical students’ self-learning or health workers’ continuing education. Traditional classroom education is currently still the main form of teaching and learning. Because online study will play a more important role in higher education, the current structure and pattern of teaching pathology will change.

Pathologists are indispensable in the diagnosis of diseases in the clinical setting. However, there is a large gap between the number of existing pathologists and the number of pathologists needed in the clinic. It is estimated that there is a shortage of 90,000 pathologists in China [2]. A similar problem also exists in the United States, Canada, and the United Kingdom [3–6]. Pathology education, especially for undergraduate students, could be the first opportunity for them to learn about the importance of pathology and the roles of pathologists. Therefore, the purpose of teaching activities is not only to transfer pathology knowledge but also to guide students to nurture robust work values.

Under the circumstances mentioned above, students’ opinions of their current pathology courses, the condition of their online study, their professional choices, and the underlying reasons for their opinions are important sources of information that can be used to optimize the current state of pathology education. However, thus far, there has been no research offering such information. Thus, we conducted this survey to extensively understand these conditions.

## Methods

Two types of questionnaires were developed and implemented for undergraduate students and graduate students (see Additional files 1 and 2). Both questionnaires addressed a variety of topics, including students’ views on the quality of traditional courses, their attitudes towards online courses, their suggestions for optimizing the courses, their experiences with online study, their professional choices, and their underlying reasons for their opinions.

Online questionnaires were administered [7, 8] to students from the Medical School of Southeast University, the Medical School of Nanjing University, and Nanjing University of Chinese Medicine. Data were collected 1 week later. Conventional statistics, classified statistics, and crossover statistics were carried out. Differences between groups were examined using the chi-square test, and the analyses were performed using Prism6 (GraphPad Software, CA, USA). *P*-values less than 0.05 were considered significant.

## Results

### Demographics of survey participants

There were 870 medical students in our sample, and the response rate was 39.31%. Among the 342 students who responded to the questionnaires, 74.85% were undergraduate students, and 25.15% were graduate students. The sample comprised 213 females and 129 males. All the participants completed pathology courses in college (see Table 1).

**Table 1:** Demographics

	Number of students	Percentage (%)
Education background		
Undergraduate	256	60.16
Graduate	86	39.84
Gender		
Female	213	62.28
Male	129	37.72
Status of course of study in pathology		
Completed	342	100.00

Characteristics of the participants.

### Suggestions for optimizing traditional pathology courses

**Fig. 1:**
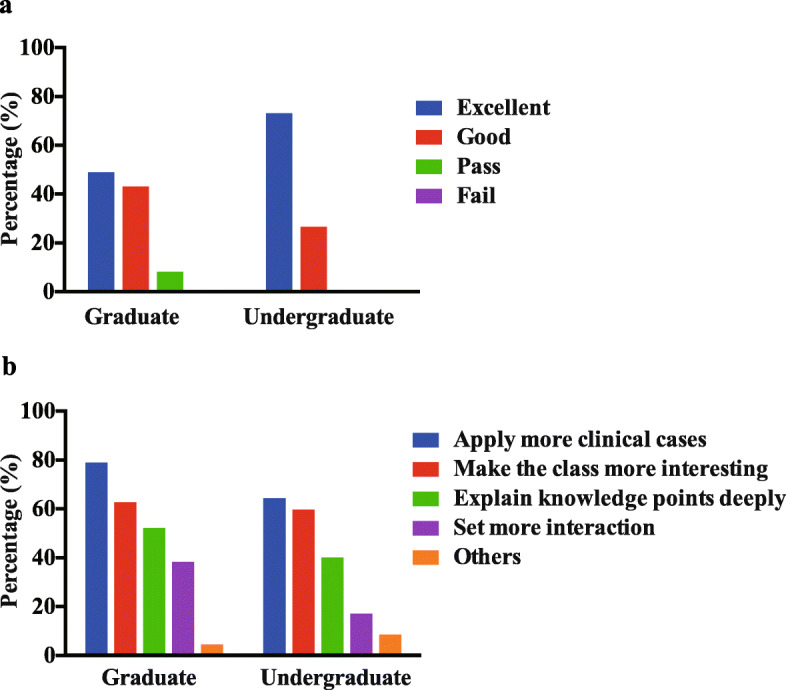
Suggestions for optimizing traditional pathology courses. **a** Students’ evaluation of the quality of traditional pathology courses. The overall quality of traditional pathology courses was classified into four levels according to the normal scoring standards: Excellent (9–10 points), Good (8–9 points), Pass (6–8 points), and Fail (< 6 points). Every student chose one option based on his/her integrative evaluation. **b** Suggestions for optimizing traditional pathology courses. This is a multiple-choice question. Students were asked to choose whichever option they agreed with. The result is the ratio of students who chose the specific option to the total number of students

As traditional courses are playing a vital role in current pathology education, we first want to know how students evaluate their quality. The overall quality of traditional courses was classified into four levels according to the normal scoring standards (the total score was 10 points): excellent (9–10 points), good (7–9 points), pass (6–7 points), and fail (< 6 points). It is shown that the majority of students are satisfied with the current courses, indicated by the total percentage of students who chose “excellent” or “good” being higher than 90.00% (graduate students, 91.86%; undergraduate students, 99.61%) (Fig. 1a).

To collect students’ suggestions on how to improve traditional pathology courses, a multiple-choice question was set, with options including teaching content, class atmosphere, and classroom interaction. The results show that the majority of students want to learn about more clinical cases based on basic knowledge, especially graduate students (79.07%) (Fig. 1b). The second-most frequently mentioned suggestion is to make the class lively and interesting. Students choosing this option accounted for approximately 60.00% of all the participants. Another important suggestion from approximately half of the graduate students is the need for more in-depth explanation of pathology knowledge. Moreover, as the results show, the interaction between teachers and students in class is popular. Other proposals with relatively low percentages include using more pictures to illustrate pathologic changes, slowing down the pace of speech, and assigning some homework.

### Students’ perception of online courses

**Fig. 2:**
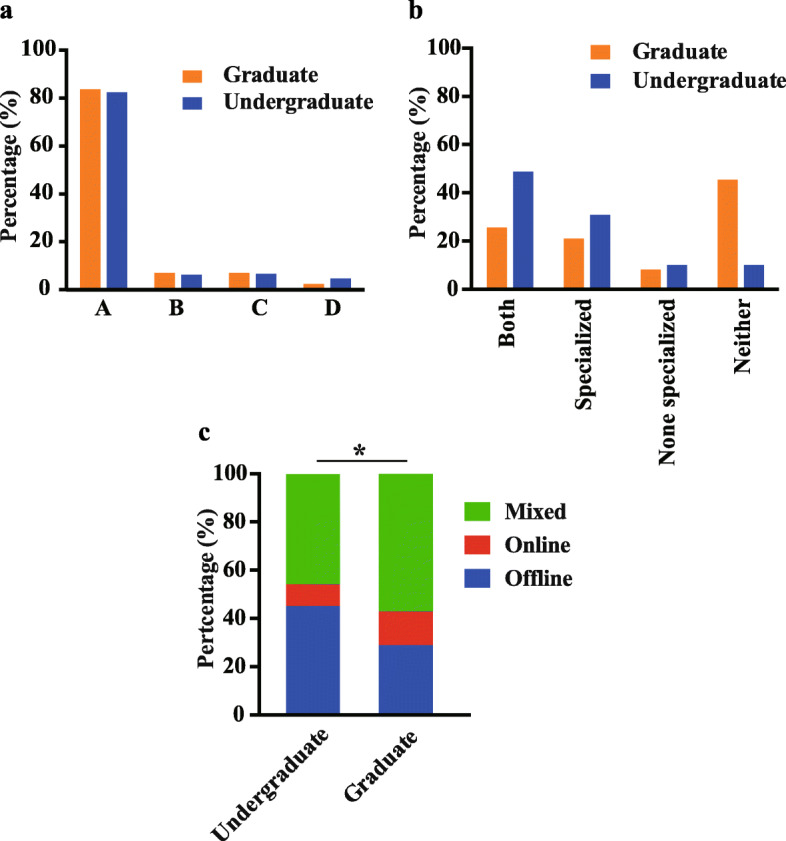
The role of online courses in students’ opinions and their learning models. **a** A, B, C, and D indicate “An important supplement to traditional courses”, “Possible to instead of traditional courses”, “Only valuable to students with insufficient offline resources”, and “Just a stunt”, respectively. **b** The question is “Which of the following online courses have you ever attended?” “Both” means “I have attended online study of both specialized courses and unspecialized courses”, “Specialized” means “I just have attended online study of specialized courses”, “Unspecialized” means “I just have attended online study of unspecialized courses”, and “Neither” means “I have not attended online study of specialized courses or unspecialized courses”. **c** The question is “If contents of online pathology courses and offline/traditional pathology courses are the same, which learning model do you prefer?”. All these questions are single-choice questions. The data are the ratio of students who chose the specific option to the total number of students. * *P* < 0.05

A large number of online courses are currently available for various subjects. Our group also launched three kinds of online pathology courses on the platform of Chinese University Massive Open Online Courses (MOOC). To understand students’ perceptions of online courses, their experiences with online study, and the kind of learning model they prefer, three single-choice questions were created. Students were asked to choose one option from the given set of answers. As Fig. 2a shows, over 80.00% of students agreed that online courses are significant supplements to traditional courses. Only a small number of students chose one of the following three options: ➀ Online courses will displace traditional courses; ➁ Online courses are only valuable to students who have no sufficient traditional teaching resources; and ➂ Online courses are dispensable.

In the investigated population, 89.84% of the undergraduate students and 54.65% of the graduate students had experiences with online study (Fig. 2b). A total of 30.86% of the undergraduate students and 20.93% of the graduate students participated in online study but about non-academic subjects. However, 10.16% of the undergraduate students and 45.35% of the graduate students had never participated in any online course.

To determine which learning pattern students like, an assumption was made to exclude any differences in the content between online and traditional pathology courses. Even in this situation, students prefer the offline model or the mixed model rather than the online model (Fig. 2c). Furthermore, the number of students choosing the online model and mixed model is larger in the graduate student group (70.93%) than in the undergraduate student group (54.68%). The reasons given by participants are that graduate students need more flexibility in their schedules, and undergraduate students need more face-to-face interactions with teachers.

### Factors influencing students’ professional choices

**Fig. 3:**
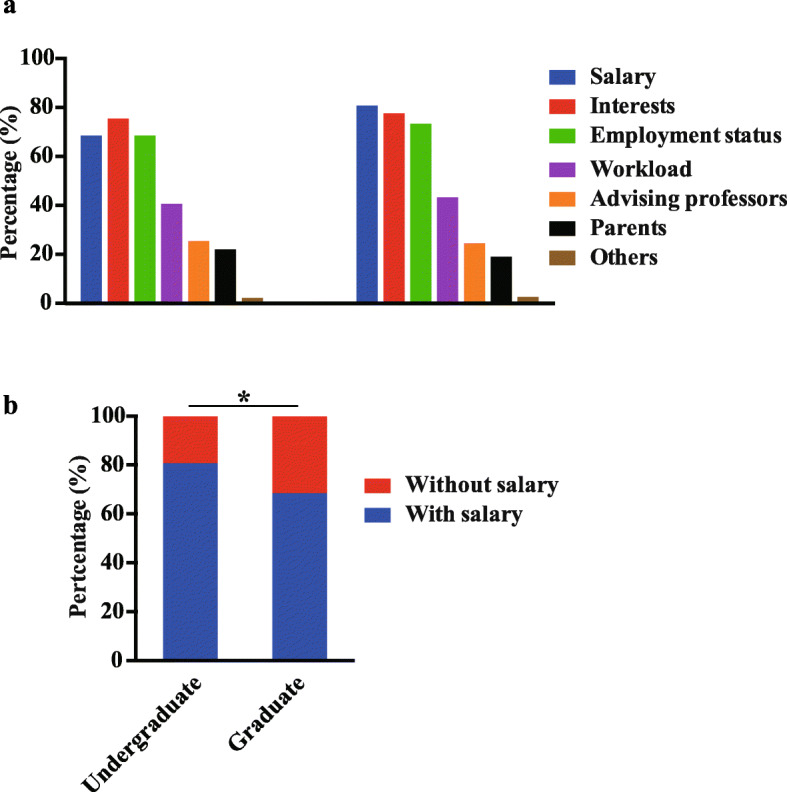
Factors influencing students’ professional choices. Students were asked to choose the three most important factors they consider when they decide their careers. **a** The result is the ratio of students who chose the specific option to the total number of students. **b** The data represent the percentage of students who chose option “Salary” or not in undergraduate students and graduate students. * *P* < 0.05

Guiding students in valuing work appropriately is an important component of teaching activities. Hence, it is important to understand what factors may contribute to students’ professional choices. We designed one question to identify the major influencing factors. For both graduate students and undergraduate students, salary, interests, and employment status are the top three factors, followed by workload, the academic position of the advising professor, the viewpoints of parents, and other individual factors. No significant difference was observed between the two groups (Fig. 3a). Interestingly, the percentage of students choosing salary was significantly higher in the undergraduate group (80.86%) than in the graduate group (68.60%) (Fig. 3b).

### The possible causes of the shortage of pathologists

**Fig. 4:**
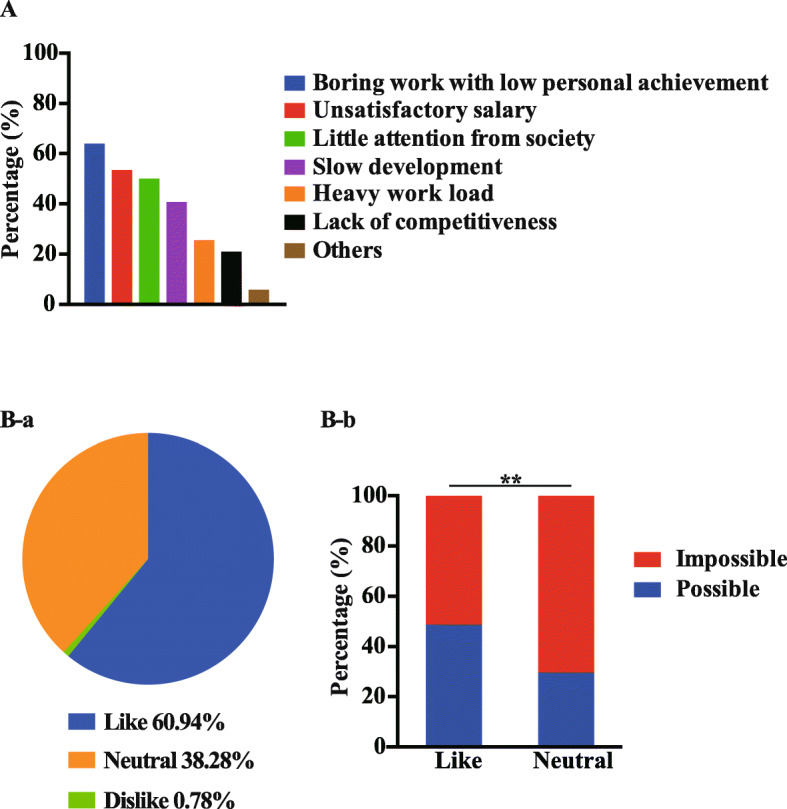
Possible causes of the shortage of pathologists. **A** The question is “What are the possible factors that cause the shortage of pathologists in the clinic?”, and it is a multiple question for graduate students. **B** These are two questions for undergraduate students. **B-a** The question is “How much do you like Pathology subject?”. Options included “To a great extent”, “To some extent”, and “Not at all”. The result is the ratio of students who chose the specific option to the total number of students. **B-b** The question is “May you become a pathologist?”. The data are analyzed by the possibility of becoming a pathologist and the emotion on the pathology subject. ** *P* < 0.01

As there is a large shortage of pathologists in the clinic, we asked the graduate students, who had decided on their specialty, what factors might have caused the shortage of pathologists. The option “slow development” refers to the underdeveloped nature of pathology departments in China. The option “lack of competitiveness” refers to the fact that pathology departments are often less competitive than other departments in the clinic. The results show that uninteresting work combined with low personal achievement is a major factor (63.95%). Low pay (53.49%) and less attention from society (50.00%) rank second and third, respectively. Notably, the heavy workload is not a major factor. Only 25.58% of students chose this option (Fig. 4A).

For the undergraduate students, a crossover analysis was conducted to illustrate the relationship between their feelings towards pathology courses and their willingness to become pathologists. As Fig. 4(B-a) shows, 60.94% of the students like pathology courses, 38.28% of students have no strong feeling, and only 0.78% dislike it. In the population of students who like pathology courses, 48.72% think that they may become pathologists. However, the possibility decreased to 29.59% in the population without strong feelings about pathology (Fig. 4(B-b)). This is in line with the result from Fig. 3, which indicates that interest has a great impact on students’ professional choices.

## Discussion

Since student attitudes have a major influence on teaching quality, their opinions on current education and suggestions to optimize it deserve special attention. This survey was designed to evaluate the quality of traditional pathology courses and their experiences with online study. Before we guide them to become pathologists, we need to determine the factors encouraging or discouraging them along this professional path.

The results of this survey clarified their suggestions for traditional curricula. The top suggestion was introducing more clinical cases. Indeed, it meets the nature of this discipline, which bridges the gap between basic medicine and clinical medicine. In fact, we tried to increase the number of clinical cases in our online course *Systemic Pathology* last year, and we received much positive feedback. Moreover, one previous study also illustrated the benefits of applying clinical case-based image portfolios in histopathology education [9]. Lively and interesting classes are widely welcomed, which was supported by another survey showing that most medical students affirm the use of humor in medical didactics [10]. Indeed, it has been confirmed that humor in class produces many advantages [11].

Online courses have the advantage of having no limitation on the number of students or the time and space of study. A large number of participants can learn in their own way at different times in various places. In particular, traditional teaching activities in college are currently interrupted in China because of the 2019 novel coronavirus disease (COVID-19). Fortunately, teachers are giving their lectures on web platforms to solve this problem. Therefore, online study is an excellent learning tool in such public events.

To integrate online courses into the current curriculum, it is necessary to take students’ study models into account. Our results show that an online-offline mixed model is the most acceptable model for both undergraduate students and graduate students. Even though the contents of the traditional and online pathology courses are the same, few students chose an online model compared with those who chose the offline model. In addition, increasing the proportion of traditional courses for undergraduate students is not ignorable, as they need more direct interactions with teachers.

The shortage of pathologists is a global problem, and it is inhibiting progress in universal health coverage [12]. It is reported that heavy workload, low pay, and the threat of lawsuits relating to improper diagnoses discourage students from specializing in pathology [13]. The results from our survey indicate that uninteresting work, low personal achievement, unsatisfactory salary, and less attention from society are noteworthy obstacles. As students who have interests in pathology are more likely to become pathologists (Fig. 4(B-b)), developing students’ interest should be one of the fundamental purposes in pathology education.

Based on these observations, we are offering a new course called *Autopsy and Accident Appraisal* to undergraduate students in our medical school. After the study of theoretical knowledge in pathology courses, students can learn the practical application of pathology in life. It is expected that they will not think pathology is boring; rather, they will think that it is interesting. We also created another new course called *Diagnostic Thinking in Clinicopathology* for graduate students. We teach the method of making a pathological diagnosis in the clinic and explain how the pathological diagnosis guides the following treatment to build up students’ interest in pathology and deliver a strong sense of personal achievement. Nevertheless, it remains to be seen whether these measures are effective.

If gender is considered, it is interesting to find that male students are more likely to attend online study than female students. In the population of graduate students, 49.15% of female participants had never attended an online course, while the percentage in the male group was 37.04% (see Additional file 3A). Another trend is observed when we analyze the main reasons why there is a shortage of pathologists. More male students chose “boring work with low achievement”, while more female students chose “less attention form society”, “slow development”, and “heavy workload”. It seems that female students are more concerned about the work environment (see Additional file 3B).

Given the small sample size of the survey, it is difficult to make a definite conclusion about how to build up a good system of online-offline courses and to determine the most important factors causing the shortage of pathologists. Another limitation of this survey is that these participants all studied in Nanjing. Nanjing is the provincial capital of Jiangsu, and medical education in Nanjing is similar to that in other provincial capitals in China. To a certain degree, these results are representative. However, Nanjing is also a developed city in China. The life situation here may influence students’ perceptions. Therefore, replication of this survey on a large scale and at multiple centers would be advisable.

## Conclusions

In conclusion, this study helps us learn more about the students. First, students are generally satisfied with traditional pathology courses. The two most important suggestions to optimize traditional courses are increasing the proportion of clinical cases in teaching content and making the class lively and interesting. Second, in students’ opinions, online courses are good supplementary materials to traditional courses, and students prefer offline-online mixed models or offline learning models to online learning models. Third, the top three factors influencing students’ professional choice are salary, interests, and employment status. Taken together, these data are valuable information for our future curriculum reform.

## Supplementary information

**Additional file 1.** Questionnaire 1 (for undergraduate students).

**Additional file 2.** Questionnaire 2 (for graduate students).

**Additional file 3.**

## Data Availability

The questionnaires and datasets used and/or analyzed during the current study are available from the corresponding author on reasonable request.

## Abbreviations

COVID-19: 2019 Novel coronavirus disease
