# Supplementary material for: A survey on the attitudes of Chinese medical students towards current pathology education
Source: BMC Med Educ. 2020 Aug 8;20:259. doi: 10.1186/s12909-020-02167-5 (PMC7414265; doi:10.1186/s12909-020-02167-5)
Supplement: Supplementary file 2 — Additional file 2. Questionnaire 2 (for graduate students). [file 12909_2020_2167_MOESM2_ESM.docx]

**Questionnaire 2 (for graduate students)**

1. Gender: [Single-choice]

| ○ Male |
| --- |
| ○ Female |

2.Status: [Single-choice]

| ○ Master student |
| --- |
| ○ MD student |

3. Why do you learn Medicine? [Multiple-choice]

| □ Interesting |
| --- |
| □ Employment status |
| □ Viewpoints of parents |
| □ Major switching |
| □ Others _________________ |

4. Have you ever heard about Pathology or pathologists? [Single-choice]

| ○ Yes |
| --- |
| ○ No |
| ○If yes, what do you know about Pathology or pathologists? _________________ |

5. How much do you like Pathology subject? [Single-choice]

| ○ To a great extent |
| --- |
| ○ To some extent |
| ○ Not at all |

6. What is the overall quality of traditional Pathology courses? [Single-choice]

| ○ Excellent (9-10 points) |
| --- |
| ○ Good (7-9 points) |
| ○ Pass (6-7 points) |
| ○ Fail (< 6 points) |

7. What do you suggest to optimize traditional Pathology courses? [Multiple-choice]

| □ Explain knowledge points deeply |
| --- |
| □ Make the class more interesting |
| □ Set more interaction |
| □ Apply more clinical cases |
| □ Others _________________ |

8. Which of the following online courses have you ever attended? [Single-choice]

| ○ I have attended online study of both specialized courses and unspecialized courses. |
| --- |
| ○ I just have attended online study of specialized courses. |
| ○ I just have attended online study of unspecialized courses. |
| ○ I have not attended online study of specialized courses or unspecialized courses |

9. If you have attended the study of specialized courses, do you think it is helpful? [Single-choice]

| ○ Yes, to a great extent |
| --- |
| ○ Yes, to some extent |
| ○ Yes, to a little extent |
| ○ Not at all |
|  |

10. If you have attended the study of online courses *Pathology and Health*, and/or *Practical Pathology*, do you think it is helpful? [Single-choice]

| ○ Yes, to a great extent |
| --- |
| ○ Yes, to some extent |
| ○ Yes, to a little extent |
| ○ Not at all |
| ○ I have not attended the study. |

11. If contents of online Pathology courses and offline/traditional Pathology courses are the same, which learning model do you prefer? [Single-choice]

| ○ Traditional/offline model, reason: _________________ |
| --- |
| ○ Online model, reason: _________________  ○ Online-offline mixed model, reason: _________________ |

12. What is the role of online courses in your opinion? [Single-choice]

| ○ Possible to instead of traditional courses |
| --- |
| ○ An important supplement to traditional courses |
| ○ Only valuable to students with insufficient offline resources |
| ○ Just a stunt |

13. What is your speciality? [Completion]

_________________________________

14. What are the three most important factors you consider when you decide your career? [Multiple-choice]

| □ Interests |
| --- |
| □ Employment status |
| □ Salary |
| □ Workload |
| □ Viewpoints of parents |
| □ Academic position of advising professors |
| □ Others_________________ |

15. What are the possible factors that cause the shortage of pathologist in the clinic? [Multiple-choice]

| □ The pathology department is often less competitive compared with other departments in the clinic. |
| --- |
| □ The pathology department is underdeveloped in China. |

□ There is little attention to the pathology department from society.

| □ Pathologists have a heavy workload. |
| --- |
| □ This job is low-paid. |

□ This job is boring with low personal achievements.

| □ Others_________________ |
| --- |
