# Supplementary material for: A survey on the attitudes of Chinese medical students towards current pathology education
Source: BMC Med Educ. 2020 Aug 8;20:259. doi: 10.1186/s12909-020-02167-5 (PMC7414265; doi:10.1186/s12909-020-02167-5)
Supplement: Supplementary file 3 — Additional file 3. [file 12909_2020_2167_MOESM3_ESM.pdf]

A

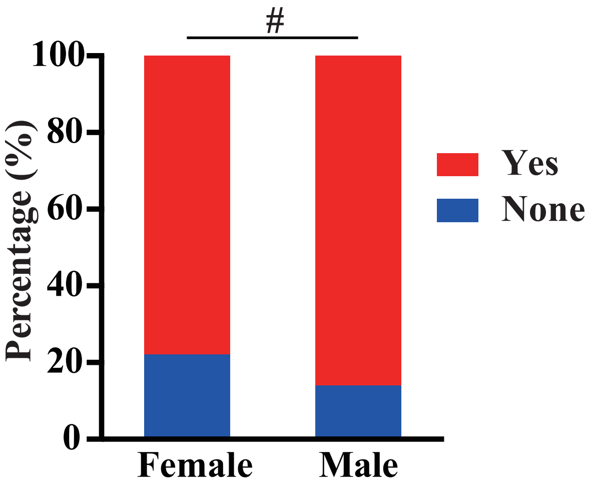


B

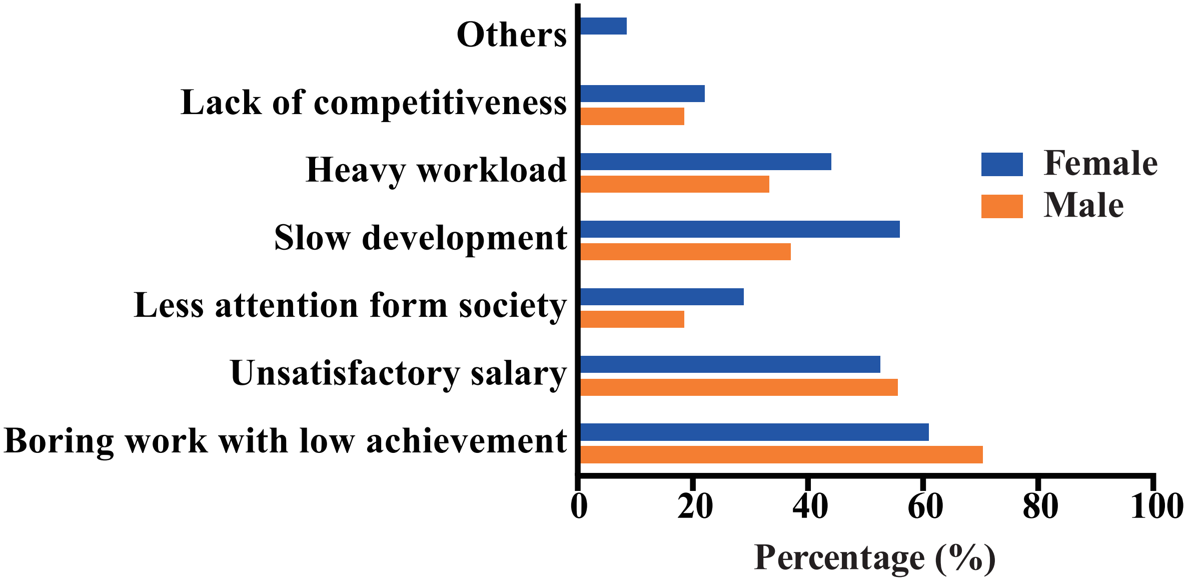


(A) The result is the percentage of students who have ever attended online study or not in female and male group, respectively. (B) The question is "Why don't you want to become a pathologist?". The result is the percentage of students who chose the specific option to the total numer of female and male group, respectively.
